# Towards an internet-scale overlay network for latency-aware decentralized workflows at the edge

**DOI:** 10.1016/j.comnet.2021.108654

**Published:** 2021-12-11

**Authors:** Pradeeban Kathiravelu, Zachary Zaiman, Judy Gichoya, Luís Veiga, Imon Banerjee

**Affiliations:** a Department of Biomedical Informatics, Emory University, Atlanta, 30322, GA, USA; b Department of Computer Science, Emory University, Atlanta, 30322, GA, USA; c Department of Radiology and Imaging Science, Emory University Hospital, Atlanta, 30322, GA, USA; d INESC-ID Lisboa/Instituto Superior Técnico, Universidade de Lisboa, Lisboa, 1000-029, Lisbon, Portugal

**Keywords:** Peer-to-peer networks, Blockchain, Edge

## Abstract

Small-scale data centers at the edge are becoming prominent in offering various services to the end-users following the cloud model while avoiding the high latency inherent to the classic cloud environments when accessed from remote Internet regions. However, we should address several challenges to facilitate the end-users finding and consuming the relevant services from the edge at the Internet scale. First, the scale and diversity of the edge hinder seamless access. Second, a framework where researchers openly share their services and data in a secured manner among themselves and with external consumers over the Internet does not exist. Third, the lack of a unified interface and trust across the service providers hinder their interchangeability in composing workflows by chaining the services. Thus, creating a workflow from the services deployed on the various edge nodes is presently impractical.

This paper designs Viseu, a latency-aware blockchain framework to provide Virtual Internet Services at the Edge. Viseu aims to solve the puzzle of network service discovery at the edge, considering the peers’ reputation and latency when choosing the service instances. Viseu enables peers to share their computational resources, services, and data among each other in an untrusted environment, rather than relying on a set of trusted service providers. By composing workflows from the peers’ services, rather than confining them to the pre-established service provider and consumer roles, Viseu aims to facilitate scientific collaboration across the peers natively. Furthermore, by offering services from multiple peers close to the end-users, Viseu also minimizes end-to-end latency and data loss in the service execution at the Internet scale.

## 1. Introduction

Future Internet, as well as 5G and 6G networks, aim to reduce Internet latency by leveraging innovations in networking research and edge environments [1]. Edge computing argues that proximity to the end-user plays a significant role in a higher Quality of Experience (QoE) of latency-sensitive services [2]. Resource sharing beyond organizational boundaries will make the edge a reality by sharing computational resources and optimizing bandwidth usage. However, computer clusters in research labs are optimized for local executions, and typically organizational policies prevent outsider access to their execution environments. An adaptive network to support collaboration across administrative boundaries at the edge still does not exist, especially for executions such as scientific workflows that require privacy and security.

The Internet is largely inefficient for heavy and latency-sensitive data transfers. The Border Gateway Protocol (BGP) [3], the exterior gateway protocol of the Internet, is not optimized for end-users. Transit and peering agreements decide how the data must go between two Autonomous Systems (ASes) [4], such as a transit provider and a content provider [5]. The Internet eXchange Points (IXPs) [6] manage these agreements, functioning as the managing entities of the Internet traffic between the ASes. Furthermore, a single data transfer has to go through ASes handled by various providers. The network transfers at the Internet are bound by agreements between the ASes rather than actual potential best paths. These factors have given rise to proposals in how the future Internet should handle the Internet traffic and how 5G networks can minimize latency in their network transfers. Content-delivery networks (CDNs) [7] minimize latency in data access by caching copies of data close to the end-users. Extending such an approach for computations will mitigate the latency incurred in workflow executions on the Internet.

Computer clusters provide a trusted distributed environment and centralized control for computations in research labs. However, computations often require more resources than those that are readily available in a local cluster. Although researchers widely use cloud environments to execute their complex workflows, cloud environments incur high latency, especially for users far from the major cloud regions and points of presence. Hence, edge environments that utilize resources closer to the users are proposed as a middle ground and the best of both worlds. Increasingly affordable cloud and small data centers have led several small-scale companies to develop and deploy various network services and other challenges ensue from the regulations in international trade limitations and complexities in the platforms for monetary transactions in fiat currency in terms of trust and flexibility remain another challenge. As such, the current cloud ecosystem does not favor new entrepreneurs who aim to offer these services to consumers at the edge.

Blockchain offers a decentralized ledger that can replace traditional service registries and transaction frameworks, providing a distributed consensus and decentralized trust among untrusted peers [8]. It extends classic peer-to-peer overlay networks with constructs for incentives and security. It features self-executable codes as smart contracts that natively support automated execution of packaged code or microservices across peers. Blockchain is used in conjunction with networking innovations such as Software-Defined Networking (SDN) [9] to improve the management of the Internet of Things (IoT) and smart buildings [10] and enhance the decentralization of sensitive applications such as healthcare [11]. However, such blockchain frameworks focus on a specific application or domain rather than providing a sandbox environment to run diverse service workflows at the edge. We envision blockchain-based decentralized frameworks facilitating fair, trustful, and flexible resource sharing while minimizing Internet latency.

### Motivation.

Given the above premises and state-of-the-art research on peer-to-peer networks and decentralized workflows, we aim at addressing the following research questions in this paper:

(*RQ*_1_) Can an innovative peer-to-peer network at the edge facilitate trustful sharing of computational resources across decentralized peers?
(*RQ*_2_) Can such an architecture solve the challenge of resource scarcity without incurring a high latency and data loss and without additional computational overhead?
(*RQ*_3_) Can we devise the executions as services and share computational resources and data across peers through service discovery over an Internet-scale overlay network?
(*RQ*_4_) Can such an approach mitigate the inequity of the Internet, reducing higher latency and data loss, both long-haul and last-mile, faced by remote Internet regions.

### Contributions.

This paper aims to answer the identified research questions. The main contributions of this paper are:

(*C*_1_) A generic blockchain framework built on top of peers as an overlay network to share Internet service instances at the edge with constructs to establish trust efficiently, with minimal data and service migration (*RQ*_1_ and *RQ*_3_).
(*C*_2_) An efficient approach to identify and model resource scarcity, latency, and data loss of the edge nodes for a latency-aware execution at the Internet-scale (*RQ*_2_).
(*C*_3_) A security protocol, Proof of Trust (PoT), as an extension to the classic Proof of Stake (PoS), to avoid incurring additional computational or resource overheads inherent to a typical Proof of Work (PoW) of Blockchains (*RQ*_1_ and *RQ*_2_).
(*C*_4_) A vision for an Internet-scale adaptive overlay network to run decentralized workflows at the edge, mitigating the high latency and data loss faced by the remote Internet regions (*RQ*_4_).

We build Viseu,^1^ an overlay framework that uses blockchain as a decentralized service registry built atop edge nodes. Viseu aims to address the challenges in executing the services at an Internet-scale in an untrusted environment. It focuses on Quality of Service (QoS) guarantees through self-enforceable and self-executable smart contracts that automate execution and incentives. Viseu uses various blockchain primitives to facilitate network service providers to provide their services and receive incentives from consumers efficiently. Viseu builds upon Ethereum [12] as its core blockchain framework by leveraging its efficient Proof of Stake (PoS) [13] scheme to ensure fairness and trust across the untrusted peers.

In the upcoming sections, we will continue to elaborate Viseu in more detail. We elaborate on the factors on the Internet that contribute to the latency and data loss and propose the blockchain-based Viseu architecture for decentralized workflows in Section 2. Section 3 presents the Viseu Viseu algorithm. We evaluate the performance of Viseu in cutting the Internet latency and data loss in Section 4. Finally, we compare Viseu with state-of-the-art and recent research in Section 5. We conclude the paper with a summary and future research directions in Section 6.

## 2. Viseu architecture

**Fig. 1.**
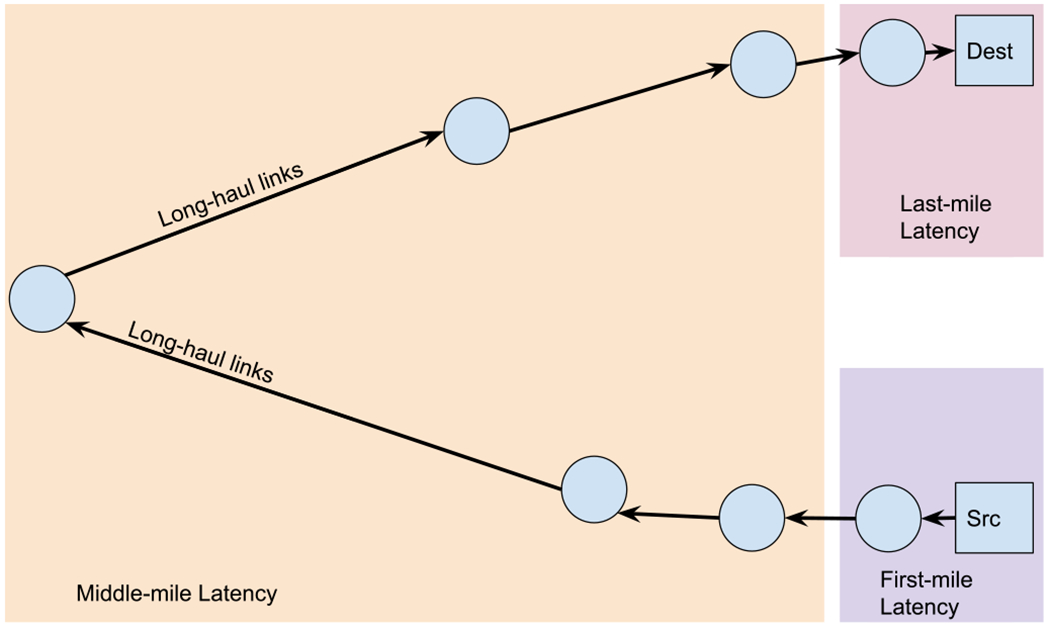
End-to-end latency at the Internet-scale.

Internet end-to-end latency between two endpoints consists of latencies incurred in the first mile, middle mile, and the last mile [14], all of which can be mitigated by utilizing the edge efficiently [1]. We build Viseu as an Internet-scale overlay network to minimize the end-to-end latency through its public blockchain architecture. Fig. 1 illustrates the three segments of an Internet path between the source and the destination of a data flow. While the Internet Service Providers (ISPs) tout higher bandwidths to their end-users, latency incurred beyond the first mile contributes significantly to the end-to-end latency and latency variation (jitter). The first-mile latency results from connecting the end-user node that operates as the source (src) to the Internet via the ISP. Similarly, the last-mile latency represents the latency resulting from connecting the destination (dest) node to the Internet. Several intermediate hops between the source and the destination nodes contribute to the middle-mile latency.

We observe that the edge environments aim at significantly shortening the middle-mile. The middle-mile often consists of long-haul links connected by IXPs [6] and transit providers. IXPs, typically located in major Internet hubs, allow interconnection between the significant ASes spanning Internet regions. Although these long-haul links are high-bandwidth fibers, any of these nodes could cause delays due to congestion. Furthermore, as the middle-mile consists of multiple ASes, the path is bound by interconnection agreements rather than the shortest routes or best paths between two endpoints.

### 2.1. Virtual internet services at the edge

We consider four critical factors in designing the Viseu blockchain architecture to share services among the peers at the edge and compose decentralized workflows from virtual Internet services.

#### Peer-to-peer resource sharing:

First, providing and consuming computational resources in a bidirectional peer-to-peer overlay network requires additional research and implementation efforts than a pre-defined resource provider and consumer model. Existing research paradigms such as volunteer computing [15] provide resources unidirectionally for a complex computational problem, rather than across peers who share their computational resources while also consuming other peers’ resources. Volunteer computing runs on the promise of altruistic users contributing their idling computational resources for a worthy trusted cause, typically of a scientific nature. However, scaling that approach to common scientific problems without well-established trust is challenging [16]. Sharing of data among peers across organizations raises privacy concerns. On the other hand, sharing computational resources to run external workflows raises security challenges in an untrusted environment. As such, sharing data or computational resources raise privacy and security concerns. The lack of trust in a large-scale network such as the Internet hinders using unknown entities’ services while also deterring users from opening up their service deployments to others due to privacy concerns. Inter-organizational networks should communicate securely to make virtual services at the edge a reality.

#### Service discovery:

Second, service discovery [17] on an ever-increasing number of service providers is a more complex challenge in federated edge environments than in the matured cloud Platform-as-a-Service (PaaS) offerings. Increasing number of small-scale edge data centers and cloud regions can host the services near to the consumers [18]. Preference for a locality-aware execution has given rise to service providers at the edge compared to the traditional cloud providers further from a large geographically distributed user base. Nevertheless, increasing services at the edge have resulted in a service discovery problem. Finding the relevant service instance is more challenging in the edge consisting of several decentralized nodes than the centralized clouds. *How do we match these service providers with the consumers that seek these services?* Signing up to and migrating the workload between various edge deployments requires additional effort, as there is no unified interface or messaging model across different frameworks. While deploying services at the edge can offer a high-performance execution for latency-sensitive Internet services, services provided virtually at the edge require a decentralized service registry to address service discovery challenges and seamless migration across infrastructures [19].

#### Infrastructure:

Third, several infrastructures must be exploited to provide an internet-scale overlay network at the edge in an economically feasible manner. Cloud-assisted networks are overlay networks built atop cloud VMs, enabling the separation of the infrastructure provider from the service provider. Pricing for cloud instances keeps decreasing, including a 51% price reduction for EC2 I3, R4, and X1 Amazon Web Services (AWS) cloud instances that are optimized for I/O- and memory-intensive applications [20]. The declining cloud prices and increasing cloud performance have steered architectures deployed across multi-cloud environments. We envision that third parties may create an overlay on cloud and data centers to offer various Internet services to the end-users with this promising future. Several research questions are left to be answered to enable a service sharing built as an overlay network. An independent and secure platform to offer a decentralized registry of these cloud-based services and a mechanism to share the service offerings between peers is still missing.

#### Rewards:

Finally, a decentralized architecture at an Internet-scale is even more challenging when the rewards are considered instead of a volunteer computing model where resources are shared for free. The rewards can be either monetary or the potential to consume services provided by other users. Blockchain [21] is a distributed ledger that offers privacy and trust among non-trusted entities, with inherent means for rewards. The cryptocurrencies enabled by blockchain, such as Bitcoin [22] and LiteCoin [23], eliminate the need for complex transactions in fiat currencies bound by local and international regulations. Blockchain extends beyond classic peer-to-peer networks such as Chord [24] and Kademlia [25] through its support for economic transactions. Blockchain architectures bring the potential for an overlay with its constructs for trust and incentives. Thus, blockchain architectures facilitate a fair and flexible market without being limited by fiat currencies’ bureaucracies and economic complexities.

### 2.2. Deployment architecture

Blockchain architectures offer scale, privacy, and freedom from a centralized governing entity. Blockchain replaces the single third-party coordinator in centralized applications with a decentralized ledger, ensuring an uncompromised execution in a large-scale untrusted environment such as the Internet. In addition to trust, accountability, and nonrepudiation, blockchain provides an economical alternative to traditional big players who operate as trusted third-party. These properties make blockchain a preferred candidate for Viseu to compose workflows at the edge by consuming the service instances hosted by the peers in their computation nodes.

**Fig. 2.**
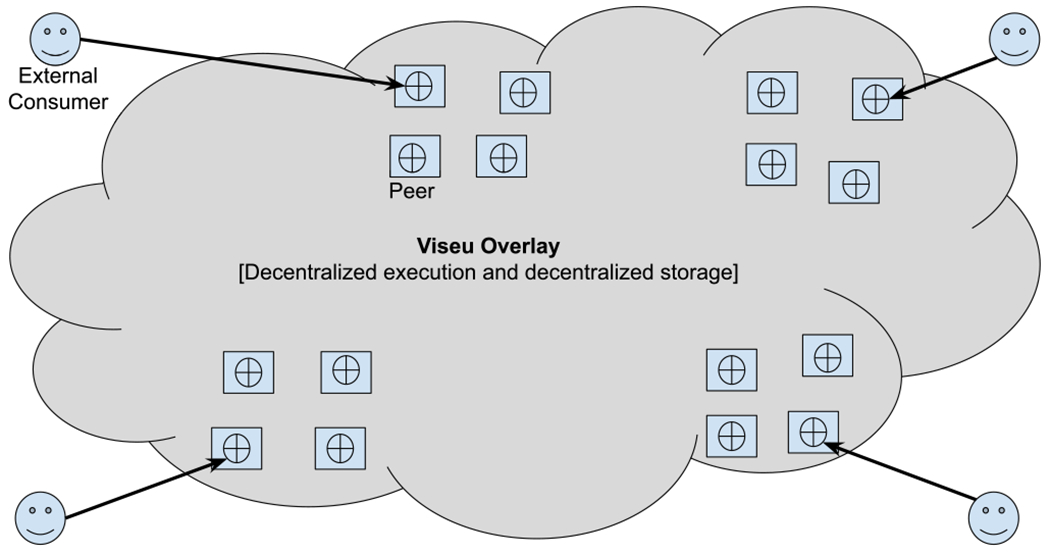
Viseu deployment architecture: Peers and external consumers.

Fig. 2 illustrates the Viseu ecosystem. Viseu consists of two sets of users: First, the peers who contribute computational and networking resources from their node to construct the overlay network; Second, the external consumers who access the services deployed in the overlay by the peers. Viseu aims to provide low latency, minimal data loss, and high bandwidth services to the peers through the overlay. It achieves the same for the external consumers of its services by letting them access and invoke the services deployed in the nodes of the Viseu overlay close to any given consumer through the overlay interface, rather than directly connecting to a physical node. The external consumers can even be mobile users or those with limited computational resources connecting to the edge, as they do not contribute their computing resources.

Each Viseu peer can be both a consumer and a provider, and they could choose to be either based on their resource availability and computation demands at any time. A peer consists of a compute node connected to the Viseu blockchain, building an overlay network at the edge. This node functions as an entry point to their relevant network service offerings. Some nodes can be thin clients (mobile devices and resource-constrained VMs), whereas others can be thick clients (laptops, servers, and clusters). The peers can share their computational resources as a service provider and also search and find services offered by others at the edge to consume.

Even when a peer has abundant computational resources, it may choose to be a consumer for two reasons: (i) if another peer hosts the desired service that is not locally available, or (ii) if the other peer holds necessary data that would otherwise require a costly, lengthy or otherwise unfeasible (privacy) data transfer between the peers. Such data transfer may not be possible due to data privacy or undesirable to avoid the communication burden of moving data to the execution. Viseu avoids sending data to the execution while also minimizing the burden of transferring executions. Thus, it adopts a “share nothing” approach as much as possible.

Viseu uses Ethereum as its blockchain due to its potential to seamlessly migrate between being a private blockchain [26] and a public blockchain. A blockchain architecture can be a public one on a large untrusted network such as the Internet, a private one with membership limited to one or more organizations, or a permissioned blockchain – a hybrid with a consortium or a trusted/semi-trusted third-party that approves new members and arbitrates the transactions. Consensus in a decentralized system is a challenge. Since there is no trusted entity in public blockchains, measures such as computational power and investment in the peers’ blockchain nodes are taken into account to reach a decentralized consensus, as elaborated in Section 3.

### 2.3. Prototype implementation

**Fig. 3.**
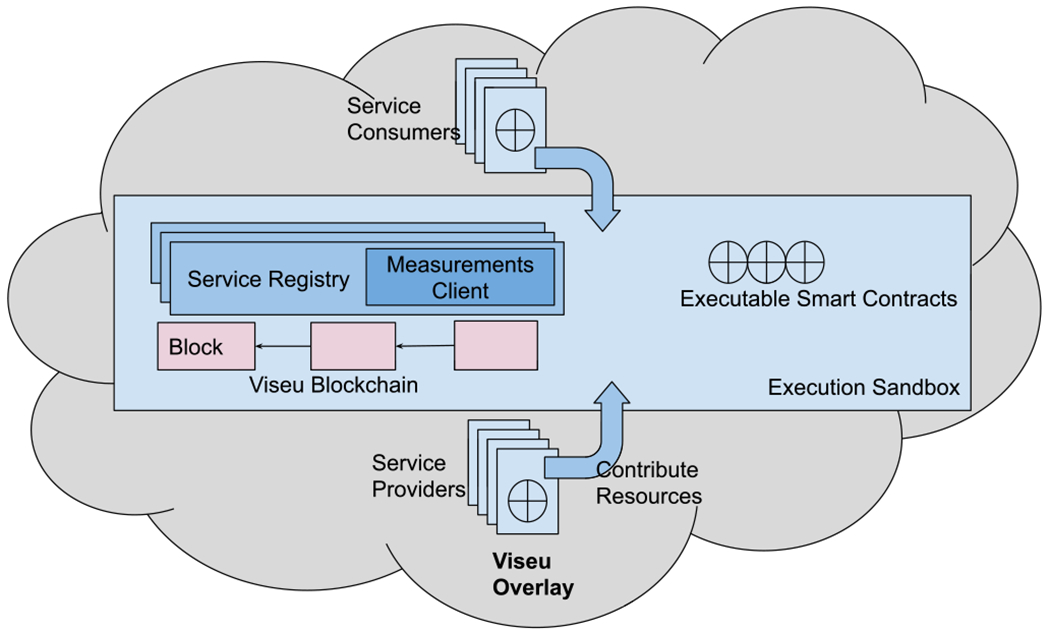
Viseu architecture and the peers.

Fig. 3 illustrates the architecture and peers of Viseu, excluding the external consumers of the Viseu services who see the deployment as a logically centralized overlay at the edge. Peers and external consumers can find their necessary service instances by invoking the *Service Registry*, a Viseu module in all the peers. The Viseu service registry is a minimal decentralized implementation of the service registry pattern [27] that consists of a *Measurements Client* for latency-aware scheduling in each peer. It offers unified access to Viseu blockchain and its overlay network that the peers can leverage as service consumers. A node can be both a service consumer and a service provider, although Fig. 3 separates the roles of the service consumers from the service providers for a cleaner notion. The blockchain is built by a series of immutable blocks, each representing the transactions and service executions.

The service registry and the ledger of the blockchain consisting of the blocks span all the peers. The measurements client incorporates network measurement frameworks into the service registry. RIPE Atlas [28] is an Internet-scale network of 11,000+ probes (small network devices) and 700+ anchors (slightly larger network devices with more capabilities than probes) connected to the members’ routers, that allows the members to measure the performance of the network and generalize the observations to the Internet as a whole. Probes are used as the source of network measurements such as ping and traceroute. Anchors can be used as either the source or the destination in the measurements. By hosting a RIPE Atlas probe ourselves over the past few years, we have accumulated more than 25 million RIPE Atlas credits (that increases by 80,000 per day), which we share across the Viseu peers for automated periodic Internet measurements by the measurements client. By default, Viseu measurements client extends and incorporates a RIPE Atlas toolkit, developed in Python, to find the low-latency peer with the necessary service instance to send the service request.

We evaluated several blockchain architectures as potential alternatives. We first considered the Hyperledger Fabric [29] as a prototype, together with the Interplanetary File System (IPFS) [30] to distribute the files to the edge on a network overlay on top of the resources shared by the peers. However, Hyperledger Fabric does not scale to the Internet’s diversity and volume as a private blockchain. We also considered Multichain [31] as it natively supports parallel executions of various workflows. However, Multichain follows a freemium model where essential features such as real-time data feeds and privacy measures such as end-to-end encryption and off-chain data purging are available only to the paid enterprise users. Ethereum [32] provides an open-source implementation of a public blockchain with interfaces developed in multiple versions. Ethereum also comes with sample smart applications that developers can extend into decentralized applications (DApps). Similar to Multichain, Ethereum also supports the parallel execution of diverse applications. Hence, we chose Ethereum as the core blockchain of Viseu.

Service providers develop executable and enforceable Smart Contracts as pointers to these services. Ethereum supports Solidity and Vyper as its programming languages to write smart contracts [32]. Viseu uses Solidity for its smart contracts. The smart contracts are executed in an execution sandbox, which ultimately sends the execution as a pointer to a microservice deployed in a peer operating as a service provider. Viseu uses Ricardian smart contracts [33] to automate the service executions and transactions at such scale and complexity while offering a verbose service description. A Ricardian smart contract is a smart contract that is carefully written to be human-readable. The smart contracts list the service offerings, along with their cost in Ether (the Ethereum currency). Proper implementation of such contracts avoids the need for a centralized entity or a mediator among the peers, providing a decentralized service registry replicated across the peers to access the blockchain and find the service instances.

## 3. Viseu algorithms

The long validation time and service discovery in a decentralized environment such as a blockchain deployment at the edge of the Internet-scale can be time-consuming. Viseu strives to minimize such overhead caused by the decentralized nature of blockchain. Viseu service discovery and PoT implementations are hence specifically optimized to mitigate these shortcomings.

### 3.1. Service discovery

Service discovery generates an initial overhead in dynamic service executions to find the appropriate service instance for the execution. Viseu follows the typical stickiness of cloud providers to ensure that subsequent service executions go to the same service instance once a service instance is discovered through the overlay. It further aims to scale with a low-overhead on bandwidth by bringing the execution to the edge, finding the most efficient nodes quicker.

Algorithm 1 presents Viseu service discovery. Each peer hosts one or more service instances that other peers and external consumers find and consume. A service can range from a simple data storage service to a radiology imaging analytics service [34]. A consumer requests to invoke a service, and the overlay maps the request to a service instance for a specific time frame and scale. The transaction completes after the task execution (or at certain intervals that serve as checkpoints in case of long-running tasks). The external consumers find the low latency service instance with the available resources through the decentralized service registry in the Viseu blockchain and invoke them directly.

**Algorithm 1:** Service Discovery

1:	**Global Variables**
2:	*peers*
3:	**end Global Variables**
4:	**procedure** Find Service Instance(ServiceSpec)
5:	id ← overlay.invoke(ServiceSpec)
6:	ComputePoT(this.id, ∅, -(*δ*_*s*_ + *δ*_*b*_) · **PoT**)
7:	ComputePoT(id, ∅, *δ*_*s*_ · **PoT**)
8:	ComputePoT(miner.id, ∅, *δ_b_* · **PoT**)
9:	**end procedure**

Viseu designs a novel hybrid protocol, Proof of Trust (PoT), to construct its blockchain. PoT extends Ethereum’s Proof of Stake (PoS) by combining it with a Proof of Work (PoW) to build trust among the peers in creating the blockchain. PoS considers a peer’s wealth measured by the balance (in the blockchain’s cryptocurrency) or the peer’s age. On the other hand, PoW defines an action performed by a peer to show that the peer is an actual node that contributes its resources to the blockchain, rather than a malicious entity masquerading as one or several nodes. Ethereum provides the means to enable execution of smart contracts, with support for incentives to those who mine the blocks [35]. The miners append the blockchain with the next block consisting of service transaction logs. These blocks construct the blockchain as an immutable linked list or a decentralized ledger. The miners get the block reward, *δ*_*b*_, a cryptocurrency or a reward in the blockchain’s overlay network, as a service fee in appending the block correctly to the blockchain.

Each execution thus cost the requesting peer the transaction to the peer that offers the service (*δ_s_* · **PoT**) and the block reward for the miner to add the block to the blockchain (*δ*_*b*_ · **PoT**). One with high PoT gets the privilege to mint the next block with transactions, earning more PoT consequently. The peer with the best service offerings that match the requested service specifications (ServiceSpec) receives the service invocation from the overlay and gets the reward for the execution from the requester.

Double-spending problems are a challenge that must be addressed in public blockchains. Double-spending refers to the scenario when a peer spends the same virtual currency twice to purchase two different resources at once due to the delay in the expense to be propagated to the blockchain’s public ledger. Viseu prevents the double-spending problem by defining the transactions atomic, recording the transactions into a block first by rewarding the honest behavior, and adding the blocks to the blockchain quickly and thus persisting the transactions with a minimal delay into the blockchain. This approach further prevents the forking of the blockchain and maintains valid and timely transactions.

### 3.2. Proof of Trust (PoT)

Algorithm Eq. (3) illustrates the algorithm to compute a peer’s PoT. The peer has the list of active peers as a global variable. That list shared across the blockchain peers is updated with the latest PoT of all the peers extending and leveraging Ethereum’s PoS consensus. The PoT of the peer is initialized with a start value of *i*, if the peer is a newly joined one. The value of *i* can depend on various properties, as a combination of PoS (such as current investment and computational resources) and PoW (such as past completed service executions). While the node is alive, the PoT is adjusted to consider the changes in these parameters.

**Algorithm 2:** Proof of Trust of a Peer

1:	**Global Variables**
2:	*peers*
3:	**end Global Variables**
4:	**procedure** ComputePoT(id, i, *δ* · **PoT**)
5:	**if** ( peers[id].PoT = ∅) **then**
6:	▷ If the PoT of the node is not initialized.
7:	**peers [id] .PoT** ← **i**
8:	**end if**
9:	**while** ( peers[id] ≠ ∅) **do**
10:	▷ While this peer stays alive
11:	**peers [id] .PoT** ← **peers [id] .PoT** + *δ* · **PoT**
12:	▷ PoT is increased or decreased based on the action.
13:	**end while**
14:	**end procedure**

Viseu chooses miners in descending order of trust. In PoS variants, the same nodes (the ones with the highest stakes) may repeatedly get to sign the next block, leading towards a *rich becomes richer* scenario through the transaction fees and minimal chance for a newcomer to mint a block. Viseu proposes a waiting period (but without influence on the stakes). A node is temporarily demoted from the pool of potential miners by marking it locally as inactive for the waiting period between two consecutive attempts to mint a block. As a fallback mechanism, when no one has enough PoS, PoT performs the next block with a PoW. However, mining is expensive. Hence, Viseu uses the service executions and the proof of executing them as a *useful proof-of-work* (uPoW). Here a miner performs the service executions, a useful task rather than SHA-256 Bitcoin-style mining. Thus, Viseu couples the uPoW (i.e., completed service executions as a reputation) and classic PoS (how much computational resources are committed to the overlay by the peer and the current investment by the peer).

The consensus in building the blockchain is an essential aspect of the blockchain framework, as blocks record the PoT of the peers while serving as immutable logs. As such, a block consisting of transactions is built after reaching a consensus among all the peers. Viseu PoT extends and leverages the existing consensus mechanism of Ethereum in building the blockchain natively with minimal overhead. Viseu also can function as a private permissioned overlay in a local network. In such a permissioned overlay, the peers are added to the overlay by the admin node manually. Hence, the PoT (or a variant of PoW or PoS) is not necessary. Ethereum’s support for both private and public blockchains allows such seamless scaling of Viseu.

Each peer (p) belongs to the set of peers (P) that build the Viseu overlay. As a dynamic overlay, peers may leave and join the overlay network at any time. Viseu does not differentiate its peers once they have joined the blockchain. Any peer node can be a miner who contributes computational resources, builds the blockchain, and verifies the transactions. PoT rewards honest behavior by increasing the chances for mining and enabling to receive the transaction fees. PoT depends on several parameters listed in (1). *Ξ* refers to how much Ether a peer owns. *c* refers to the computational resources that a peer committed to Viseu. *R* refers to the past performance of the service instances of each peer. It represents over time how the service instances of a peer faired, adjusted for the weight and length of the service offerings to minimize those with smaller but multiple offerings rank higher than those who have offered a much more significant but lower number of service instances.


(1) $$\begin{array}{l} \Xi \Rightarrow \text{PoS}:\text{Current investment of the peer in Viseu}.\\ c\Rightarrow \text{Committed computational resources}.\\ R\Rightarrow \text{Reputation}–\text{past successful service executions}. \end{array}$$

Eq. (2) shows the total available computational resources (C) of the overlay at any given time. Here, c is a compound variable of computational resources, memory, processing power, and storage.


(2) $$C=\sum_{\forall p\in P}c.$$

Viseu defines trust as a function of *Ξ*, c, R. *t_i_* and *t_f_* refer to when a peer joined the Viseu blockchain and the current time, respectively. PoT assumes a cumulative value for trust, which can be represented by a time integral. This cumulative value rewards those with a long time maintaining a positive trust, as Eq. (3) shows.


(3) $$PoT=\int_{t_{i}}^{t_{f}}f\left(\Xi ,c,R\right)dt.$$

**Fig. 4.**
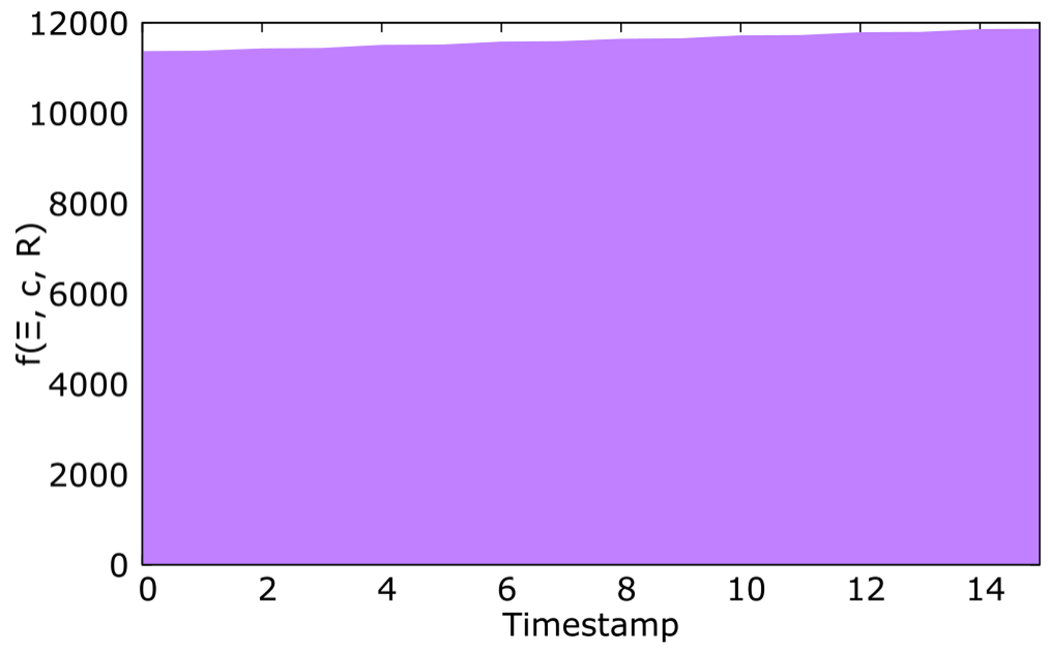
Proof of Trust (PoT) as a time integral.

We elaborate this notion of PoT with a series of executions on a Viseu peer. Fig. 4 illustrates a simplified sample representation of PoT. Here, *t_i_* = 0, *t*_*f*_ = 15. The PoT starts with a high value due to the committed computational resources from the node (c) and the current investment as PoS (*Ξ*). However, there is no reputation (R) from previous executions since the peer is new. Several executions were performed on the blockchain during the considered timeframe, changing the value of *Ξ*. Computational resources that the peer committed to the Viseu overlay remain constant during the timeframe, as is most often. Hence, f(*Ξ*, c, R) can be reduced to c * f(*Ξ*, R). Viseu favors long-term peers to prevent one with a bad reputation from disappearing and reappearing with a new identity.

Following the classic blockchain approach, Viseu deterministically chooses a peer as a minor to sign and mint the next block from a pool of nodes with a high PoT to achieve distributed consensus. Anyone with a higher PoT may challenge the miner’s decision in minting the block in case of malicious behavior. Viseu further envisions a reduction in PoT to promote correctness. Therefore one does not risk losing their reputation for the sake of acquiring the transaction fees for one block. Viseu stands as its own custom Ethereum deployment. It does not possess the capability to convert to/from fiat currency to its own Eth. As with any blockchain, with more nodes, Viseu becomes more secure as the 51% attacks (gaining control of the majority of the peers constructing the blockchain) will not be feasible with a large number of peers.

A transaction consists of the lease payment from the service consumer to the provider and the details on the consumed services. The cost of a service offering in the cryptocurrency is defined as ‘gas’ in Ethereum. Upon completing the lease time, task completion, or at time intervals as batches as specified in the smart contract (for example, for long-term on-going jobs or expensive tasks), the transaction is initiated, and the gas is transferred to the provider. Such a transaction increases the *Ξ* of the provider and decreases the same from the consumer.

The providers define a fraction of the lease payments as the transaction fees in the smart contracts, which the miners will take as the reward for including the transaction in the next block and minting the block into the blockchain. Providers are incentivized to offer a higher fraction of transaction fees to attract the miners/accelerate the chances of inclusion in the next block. On the other hand, the transaction fees cannot exceed the revenue a service provider acquires from executing the services for the Viseu blockchain to be functional and stable.

## 4. Preliminary assessments

We assess if an overlay for virtual Internet services is feasible and enables cutting the Internet latency and data loss. We then benchmark Viseu against the base network for service executions at the Internet scale.

### 4.1. Latency and data loss in the last-mile

We first build a case for Viseu overlay spanning a wide area network or a metro area network, but for external consumers at Internet-scale, by measuring last-mile latency and data loss that could typically be avoided by a decentralized service execution from any node (or several nodes) of the network. We performed traceroute and ping measurements from RIPE Atlas probes to our server nodes in Lisbon, Portugal, and Atlanta, GA, USA. We then performed traceroute and ping measurements between pairs of Atlas probes and anchors. We made several observations where the last-mile contributed significantly to high latency and data loss.

**Fig. 5.**
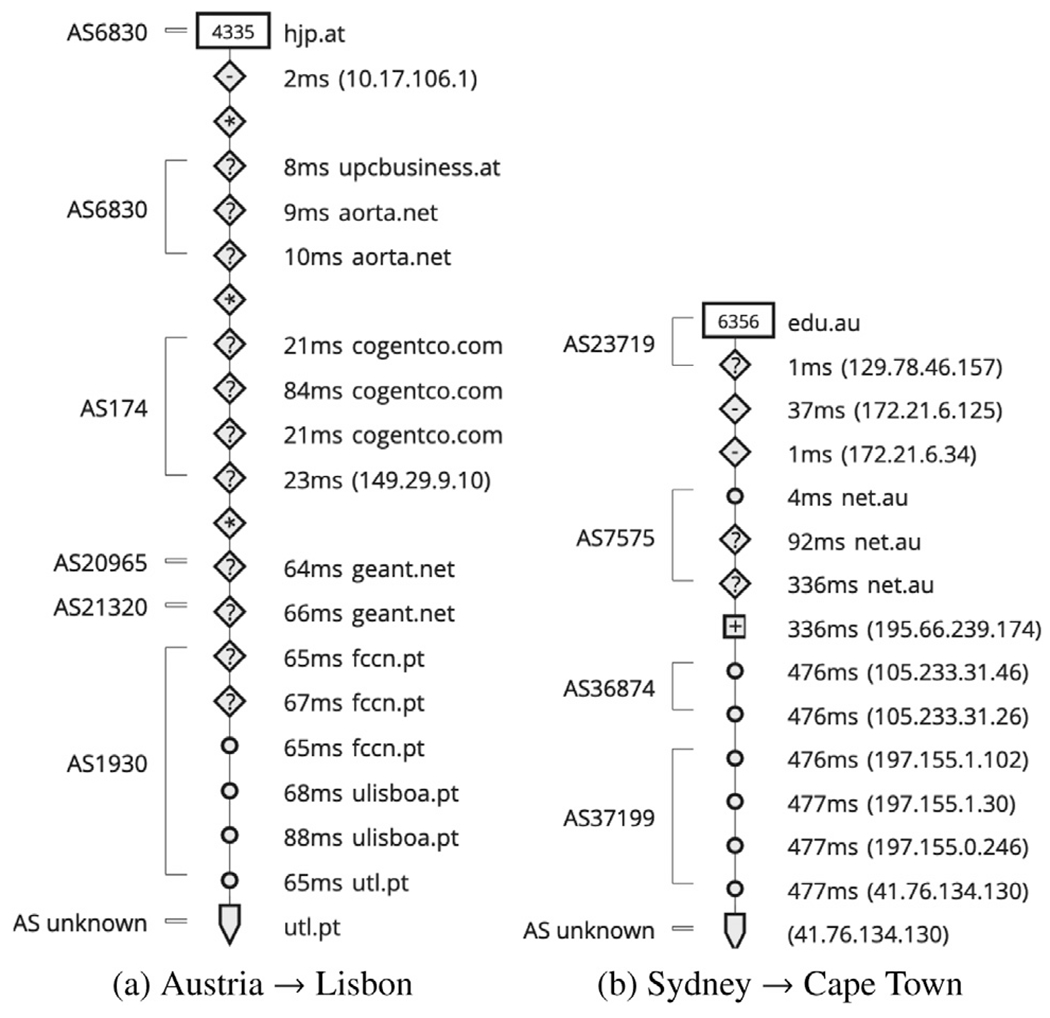
Understanding the last-mile latency through traceroute.

**Fig. 6.**
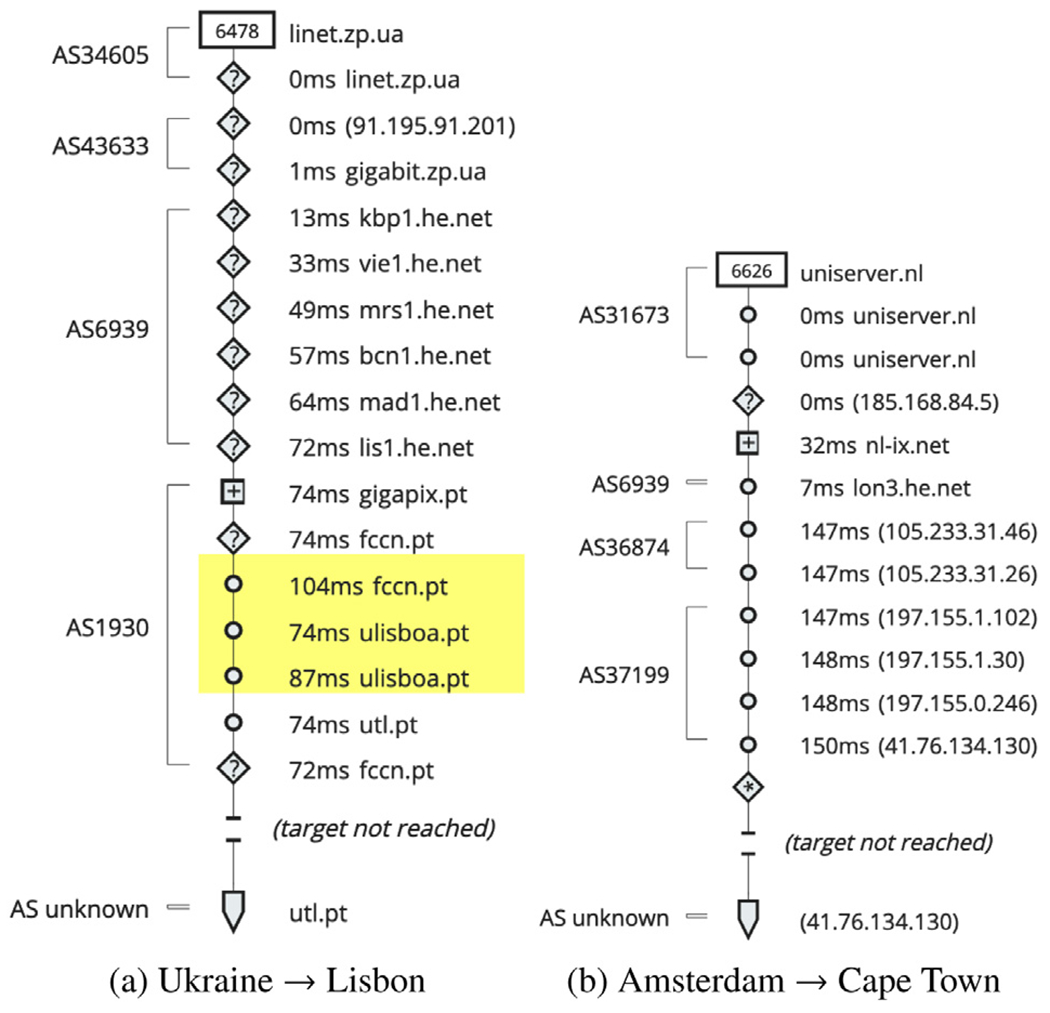
Understanding the last-mile data loss through traceroute.

Fig. 5 illustrates two of our sample measurements where the middle mile and first mile have a significantly low latency than the last mile in both observed cases. Similarly, Fig. 6 illustrates two other sample measurements where the data loss occurred in the last mile. Since a data loss would indicate an unsuccessful attempt of data transfer, it could not be used to measure end-to-end latency. Hence for the two sets, one pair is for last-mile latency and the other for data loss. To provide a more inclusive representation for both cases, we include two end-to-end transfers for each case. The first represents a shorter distance, whereas the other is an inter-continental data transfer that leverages long-haul Internet backbone networks. We also note that long-distance does not always translate into a higher number of hops due to a major connectivity provider connecting two endpoints spread across continents.

Fig. 5a illustrates end-to-end latency from a probe from Austria to the server in Lisbon, Portugal. The network flow passes through the AS in Austria (AS6830) and international connectivity providers (Cogent – AS174 and Geant – AS20965 and AS21320) before reaching AS1930 in Portugal. The autonomous system (AS) AS1930 is managed by FCCN (FCT - Unidade de Computação Científica Nacional), the scientific computation unit of the Foundation of Research and Technology - FCT, which is a Portuguese education network. AS1930 incurs a significant latency, despite being entirely located in Lisbon, Portugal, close to the destination. Fig. 5b shows a similar observation for a traceroute from a probe in Sydney, Australia, to an anchor in Cape Town, South Africa. In this case, the intercontinental latency from Australia to Africa incurred less latency than the last mile. The last two ASes (AS36874 and AS37199) are entirely in Cape Town, South Africa, but incurred more latency than the first-mile (AS23719 and AS7575 – the ASes in Australia) and middle mile consisting of the IXP in the UK.

Fig. 6 illustrates two more of our sample measurements where the traceroute did not succeed. Here, in both cases, we observe that the last mile contributed to data loss. Fig. 6a shows data loss after reaching the FCCN network in the last mile in Lisbon, in a traceroute from Ukraine to Lisbon, Portugal. The flow transfers through two ASes of Ukraine (AS34605 and AS43633), reaches a transit provider he.net (AS6939) in the UK, reaches an IXP in Lisbon, finally reaches the FCCN network (AS1930), but eventually fails to reach the destination. Fig. 6 similarly shows data loss in the last mile in Cape Town, South Africa, in a traceroute from Amsterdam, Netherlands, that passes through several ASes. Its path includes the first mile in the Netherlands (AS31673), an IXP in Amsterdam, Netherlands, he.net (the same transit provider, AS6939, as before), before reaching the ASes of Cape Town (AS36874 and AS37199), but still not reaching the target.

We note that an overlay spanning the wide area network of FCCN would have eliminated the high latency in Fig. 5 and data loss in Fig. 6. As this is a purely educational network, deploying Viseu as a blockchain framework for decentralized and replicated service instances to serve the requests from anywhere in the overlay, rather than a specific node, would have prevented such last-mile latency and data loss. We make the same observation with the latency and data loss relating to Figs. 5b and 6b. An overlay deployment across the Cape Town metro area network to respond to the external consumers’ service requests would significantly reduce such latency and data loss incurred in the last-mile.

### 4.2. Internet latency and latency variations

**Fig. 7.**
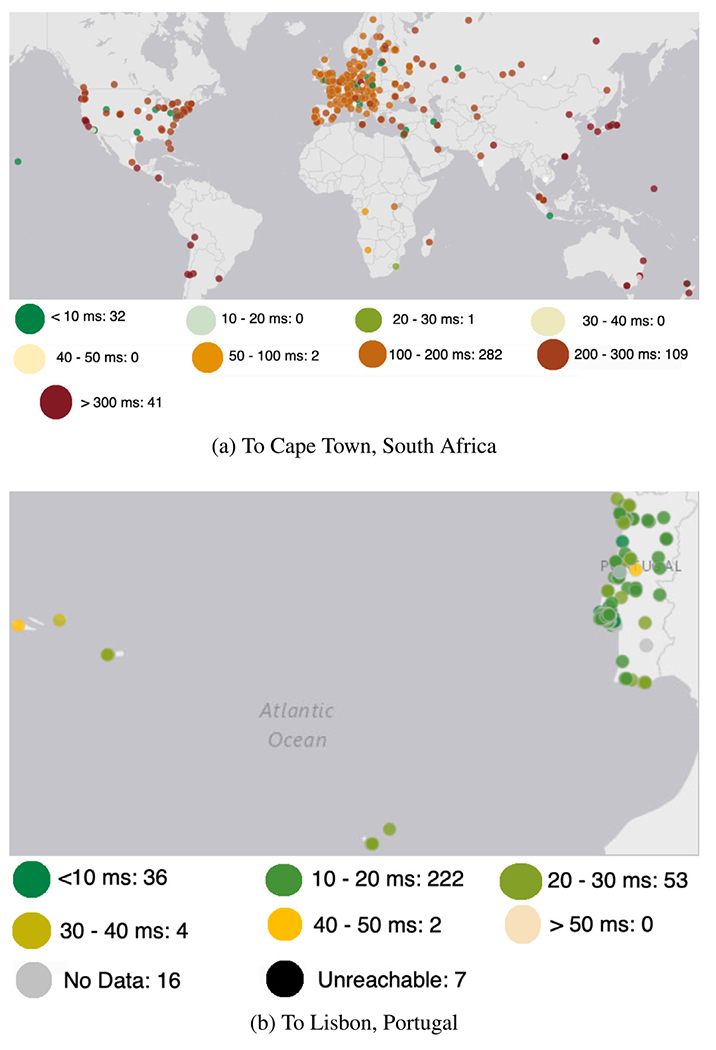
Latency variations, from various probes.

We then illustrate the network latency and its dynamic variations between pairs of Internet endpoints using RIPE Atlas to demonstrate the need for a measurements client for Internet-scale service scheduling. We observe the Internet latency and how physical proximity performs a role in it with ping measurements. We demonstrate the contributions of Internet latency by measuring latency across several RIPE Atlas probes and anchors. We visualize two of our observations with RIPE Atlas. First, latency from various probes across the globe to a probe in Cape Town, South Africa (Fig. 7). We also observe the latency from multiple probes in Portugal to a probe in Lisbon, Portugal (Fig. 7). Both figures show the range of end-of-end latency and the number of occurrences (i.e., the number of pairs of nodes with the specified range of latency).

We observe that simple geographical proximity does not always translate directly into a low latency or fewer number of hops, both at the Internet-scale (shown by global measurements in Fig. 7) and a national scale (demonstrated by the measurements in Portugal in Fig. 7). Major Internet hubs often incurred low end-to-end latency, even though they are far from the destination (from Cape Town and Lisbon, respectively), due to the reliance on the so-called Internet fast-lanes provided by connectivity providers such as transit providers.

**Fig. 8.**
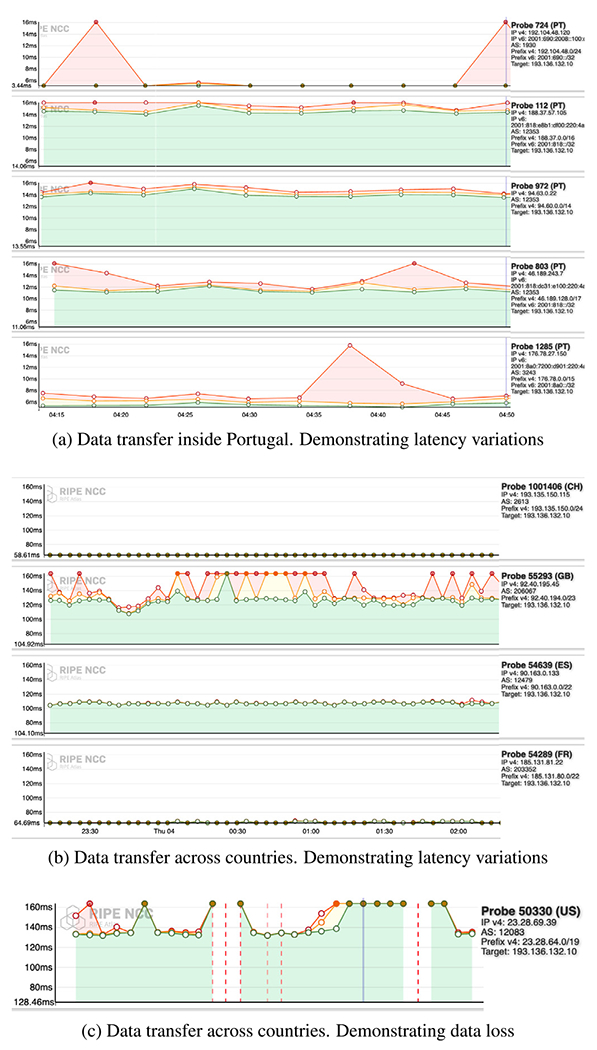
End-to-end latency variations with time to Lisbon.

Even the same Internet node may have variations in latency and data loss with time. We use latency variations through ping measurements over time, to probes in Lisbon, from probes in Portugal and worldwide. Such variations occur even for packets sent in parallel at the same time, as shown by Figs. 8a and 8b. Fig. 8c demonstrates data loss across a pair of RIPE Atlas probes at times. While we measure the Internet latency and data loss over time with measurement tools such as RIPE Atlas, BGP is not optimized for low latency or best paths. Hence, an overlay and a dynamic measurements client of the Viseu avoids such high-latency peers in real-time by leveraging its blockchain-based decentralized service registry.

We posit that distributing the clients effectively at the edge in such a network can eliminate delays in accessing the services hosted in distant destinations as the first mile. Similarly, using Viseu to run the services from any peer (or several peers) in an inter-domain overlay network (such as the one that spans FCCN, which covers many research institutes and university networks of Portugal such as ulisboa.pt and utl.pt) will mitigate such latency to both the overlay peers as well as the external consumers from the remote Internet regions. With its efficient decentralization of data over an overlay at the edge, rather than a single end node, Viseu can prevent the last-mile latency and last-mile data loss that we observed.

### 4.3. Viseu prototype deployment

We deployed a Viseu prototype on six servers in a research cluster and across 22 VMs spanning across all the 21 AWS cloud regions available as of spring 2021 (Bahrain, São Paulo, Stockholm, Paris, London, Milan, Ireland, Frankfurt, Canada, Tokyo, Sydney, Singapore, Seoul, Osaka, Mumbai, Hong Kong, Cape town, Oregon, North California, Ohio, and North Virginia). We used 21 VMs - one per cloud region as worker nodes in the public blockchain deployment of Viseu. We configured Viseu as a permissioned blockchain to enable communication with our local research cluster in Atlanta. In the permissioned blockchain, we used 6 VMs in the research cluster and the 21 cloud VMs, thus making 27 worker nodes. We used one node in North Virginia as an admin node that manages the admission to the Viseu as a permissioned blockchain.

**Fig. 9.**
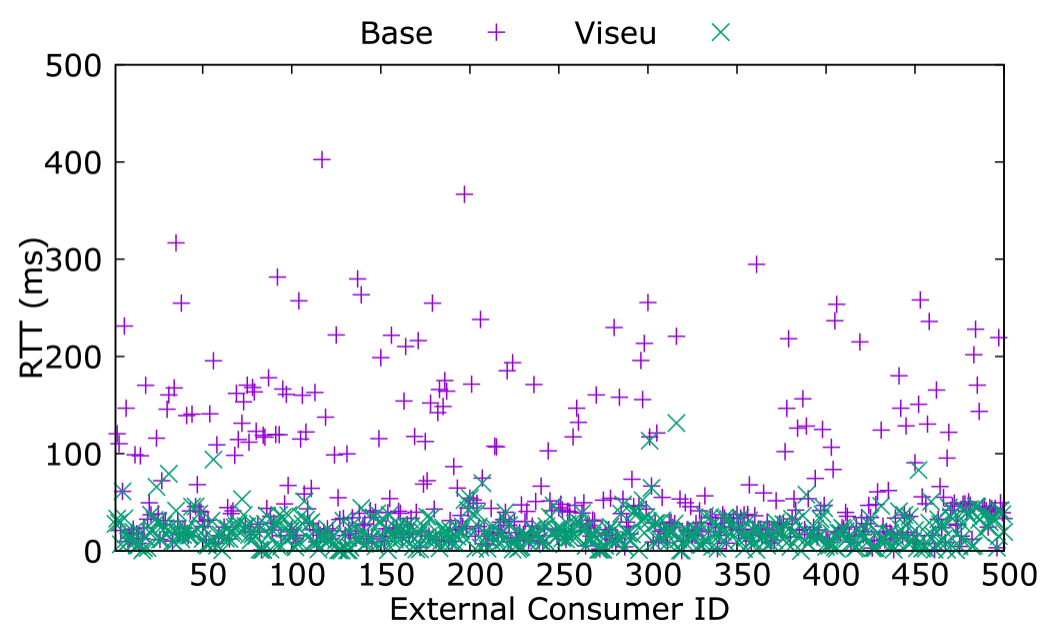
RTT with a stand-alone node vs. Viseu.

We evaluated the performance and efficiency of Viseu in minimizing the Internet latency, data loss, and the number of hops through its overlay, compared to a single server hosted in a cloud VM in Frankfurt offering the service as the base. We emulated 500 external consumers to measure the performance, using 500 RIPE Atlas probes spread across the globe. Fig. 9 benchmarks the latency of Viseu overlay for an external consumer against the base. Each external consumer is referred by a unique identifier that we call the “external consumer ID”. The latency is measured as a round trip time of 16 packets, each with 2048 bytes of data (the maximum values that RIPE Atlas can transfer).

We chose Frankfurt to deploy the base (rather than other cloud regions) as Frankfurt is a central cloud region located in a major Internet hub. Our preliminary assessments also indicate that cloud VMs in Frankfurt provide the least latency, number of hops, and data loss when accessed from across the globe, compared to the cloud VMs with a similar network interface from the other regions. We observe that we can significantly minimize the end-to-end latency in service executions by building an overlay that spans the Internet-scale while utilizing the computational resources at the edge close to the service consumer.

**Fig. 10.**
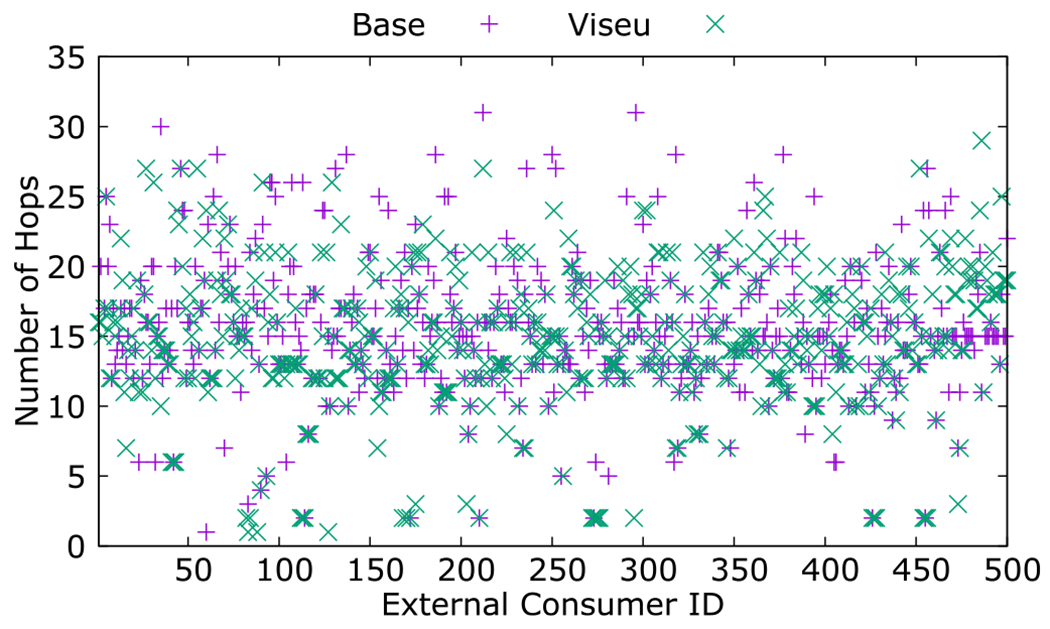
Number of hops with a stand-alone node vs. Viseu.

We benchmark the number of hops for the same set of executions for Viseu against the base, with traceroute measurements. We observe that Viseu typically reduces the number of hops while reducing latency, as illustrated in Fig. 10. Physical proximity and the number of hops contribute to end-to-end latency and how Viseu routes the data transfers across its overlay network. As Figs. 5 and 6 indicate in their sample traceroute paths, physical proximity does not always translate into the number of hops. Since latency does not merely rely on the number of hops, sometimes Viseu uses more hops between the source and the destination nodes, as the overlay is built for low latency.

**Fig. 11.**
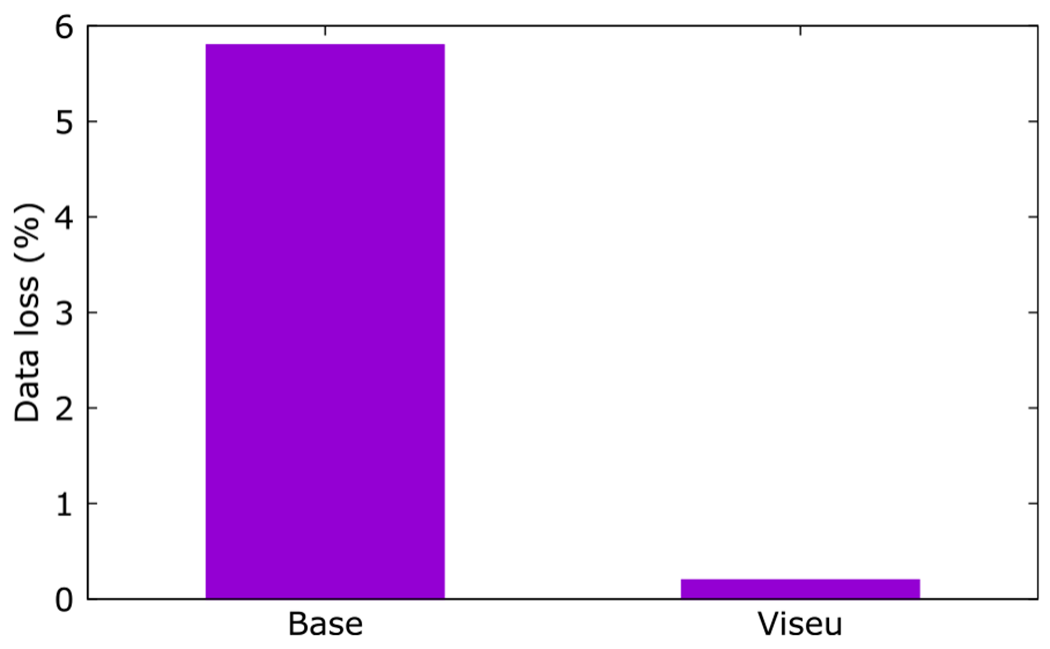
Data loss with a stand-alone node vs. Viseu.

Fig. 11 compares the data loss with Viseu against the base. We notice that 5.8% of the packets were lost with the base for just one attempt of sending the packets. With Viseu overlay, the data loss dropped to 0.2% with a single attempt of sending the packets, a nearly 30-fold reduction in data loss.

Our preliminary assessments highlight that decentralized workflows at the edge can significantly reduce latency and data loss while also minimizing the number of hops that a network flow must travel before reaching the destination. We note that Viseu can facilitate a decentralized architecture for efficient decentralized data storage and service executions, thus minimizing last-mile data loss and latency for external consumers while also providing minimal latency, data loss, and the number of hops for the peers. We note that an adaptive overlay network at the edge will mitigate the high latency multiple hops incur by providing decentralized computational resources and storage for executions.

## 5. Related work

### 5.1. Resource sharing in wide area networks

Idling compute resources have been used for various computation-intensive tasks in volunteer computing projects such as BOINC (Berkeley Open Infrastructure for Network Computing) [36] and SETI@Home [37]. While volunteer computing distributes and executes tasks in parallel, it lacks an incentive mechanism for those who contribute their resources (even when users are allowed to submit their own projects for execution in the community [16]). An incentive mechanism is essential to facilitate a decentralized execution of commercial applications and research works that do not attract sufficient popularity to receive resource contributions through a volunteer computing model.

Blockchain architectures enable resource sharing with incentives and trust. There has been research on how to incentivize the workers fairly without encouraging dishonest behavior for higher rewards [38], and rewards with trust in volunteer computing and crowd sourcing [39]. Golem [40] is made of users who either provide or consume computing resources, thus creating a decentralized supercomputer. iExec [41] is a distributed cloud platform for decentralized applications (DApps) that offers RLC cryptocurrency as tokens. SONM (Supercomputer organized by network mining) [42] is a framework similar to Golem and iExec, which provides services and use cases such as game servers, scientific research, and machine learning. It sought to address these challenges by verifying the correct execution through smart contracts. SONM uses an Ether-based own currency as a reward to the resource providers. SONM has challenges similar to that of Viseu on ensuring the correct execution of the services. However, Viseu posits the blockchain framework to exploit the edge to mitigate end-to-end Internet latency and data loss.

Previous research has proposed blockchain-based payments for services deployed at the edge or the fog. Etherum-based payment is used in research for fog services instead of using a centralized third-party payment gateway while also providing measures for trust [43]. Research has also proposed using Bitcoin to reward the workers in fog computing, with the presence of a semi-trusted third party for arbitrations [44], and in edge computing scenarios integrated with energy efficiency concerns [45]. Traditionally, the rewards have been paid through a fiat currency, potentially through a trusted intermediary to resolve conflicts. However, such an approach incurs service costs by the intermediary and the fiat currency’s administrational challenges and transaction costs. Due to its decentralized nature, blockchain eliminates the need for a centralized managing entity. The lack of a centralized managing entity saves the intermediary costs while enabling a secure overlay network among untrusting peers. These works are similar to Viseu. However, Viseu aims to use blockchain more as a peer-to-peer overlay for resource sharing beyond a local area network than a payment system.

Distributed file systems reduce the network latency in file access by storing files at the edge, close to the user, following an approach similar to Viseu. Previous work has used blockchain for decentralized transfer learning [46]. Substratum [47] distributes and stores content in the peers’ servers, thus aiming to achieve a decentralized web. Sia [48] is a decentralized cloud storage backend that splits, encrypts, and distributes user files in a decentralized network. Only the file owner can access the file with the key, despite its distributed and decentralized nature. Sia provides a higher cost-efficiency when benchmarked against S3, Google Cloud, and Azure. Filecoin is a blockchain-based decentralized storage network [49]. Viseu focuses on providing a distributed computational environment to execute services with minimal data transfer rather than providing decentralized storage. However, it can complement a decentralized file system such as IPFS for quick file access from the edge.

Smart contracts provide means to automate scripts that run in the decentralized nodes of the blockchain. OpenBazaar [50] uses human-readable smart Ricardian contracts similar to Viseu. But its smart contracts focus on automating the payment and liability of the parties involved, whereas Viseu’s smart contracts enable the service execution in the peers. Smart contracts have been used to ensure SLAs from cloud to edge environments [51] and to support QoS-aware service compositions [52]. Viseu shares the approach and scope with these frameworks through its use of smart contracts and the focus on SLA and QoS for service composition workflows at the edge. The Internet Blockchain [53] functions as a backbone for the Internet by offering distributed, tamper-resistant transactions. As future work, we aim to deploy Viseu as a permissioned blockchain at the Internet scale, hence complementing the Internet Blockchain and the other state-of-the-art.

### 5.2. Proof of Work (PoW) and alternatives

Various alternatives have been implemented to build the blockchain from the blocks consisting of transaction data. These approaches focus on combating spam and eliminating cheating, such as the double-spending problem. PoW is implemented in bitcoin and many other blockchain frameworks, where peers (or miners) resolve (with a potential reward) a computation-intensive challenge that is easier to confirm. Thus, PoW aims to reach consensus among peers by making it expensive for malicious peers to compromise the system (except by owning 51% of the computational resources in the blockchain). Typical PoW wastes computational power and energy as these hash-based computations are useless except for securing the blockchain. Hence, various alternatives have been proposed. Proof of Ownership (PoO) enforces mining only through specific trusted execution environments (TEE) [54]. TEEs offer guarantees for execution and also link execution to a processor. By coupling the identity to a single processor, TEE makes it expensive for a malicious user to attack the blockchain without owning 51% of the physical computing resources of the entire blockchain, thus minimizing the potential for Sybil attacks.

Proofs of Useful Work (uPoW) [55] replaces the typical PoW in blockchain with some valuable computation for the scientific, mathematics, or computational community or to the framework itself. These computational problems include Orthogonal Vectors (OV), 3SUM, All-Pairs Shortest Path, and any graph property stated in first-order logic. PrimeCoin [56] finds particular kinds of prime numbers that are of interest to the mathematics community. Cuckoo cycle [57] follows a graph-theory-based PoW in finding specific cycles in graphs. PermaCoin [58] expects the miners to offer distributed storage for archival data, thus minimizing the computing resource wastage. Unlike PrimeCoin and Cuckoo cycle, PermaCoin is of direct interest to the platform. Viseu uses its service invocations as a uPoW, as a component of its PoT, in conjunction with PoS.

Proof of Stake (PoS) is an energy-efficient alternative to PoW, as it replaces mining with the stake or how much someone has invested in the blockchain. PoS gives the right to mint the next block to one with a high stake (more coins/stakes/investment in the blockchain). The one who mints the next block receives the transaction fee included with the transactions. Ethereum [59] and PeerCoin [60] are two projects that use PoS. PoS is extended to consider more constraints to address shortcomings, such as the potential for the same entities to receive minting rights repeatedly. For example, coin-age (duration/age of the stakes) is considered in minting and resetting the coin-age once someone mints the next block. Peercoin resets the coin-age once a PoS is used to sign, forcing a 30-day wait between another attempt to sign the blocks by the same node. Ethereum is switching to PoS from its previous PoW implementation by default [61]. Viseu uses PoT, a PoS variant, as its primary mechanism for verifying nodes and transactions in its public blockchain.

Proof of Burn (PoB) [62] is a variant of PoS that “burns” some coins by sending them to an unspendable invalid address. PoB is used as either a way to bootstrap a new currency or as a replacement for PoW in mining – for example, Counterparty [63] alternate coins were distributed for those who burned some bitcoins. Slimcoin [64] starts with PoW in its early stage and switches to PoS and PoB once an initial number of coins are created as block rewards. Anyone can burn a few of their coins to secure the following mining rights in Slimcoin. Sidechains are where a user sends a certain amount in a currency (can be bitcoin) to a specified account in another blockchain (typically a new one) and receives the equivalent amount of new currency. Contrary to PoB, two-way transactions between the old and new currencies are possible as in a money exchange. In PoB, burned coins are lost forever and cannot be recovered.

Research approaches including Viseu consider a combination of PoW (often, a uPoW) and PoS, as resources are scarce in research and cannot be wasted as in bitcoin. Gridcoin [65] consists of a PoS and a Proof of Research (PoR) algorithm to secure itself. Its BOINC-based PoR replaces the wasteful typical PoW schemes. It uses BOINC APIs to infer how much resources have been contributed to BOINC by a node. Thus the PoR contributes cycles to the volunteer computing to do actual scientific research work. Proof of Activity [66] makes PoW less wasteful by coupling the PoS with the miner’s activity, as a combination of PoW and PoS. Proof of Activity is similar to the Viseu’s PoT implementation as both consider a combination of PoS and PoW.

## 6. Conclusion

Sharing services and computational resources across multiple organizations is challenging. The cloud ecosystem and Internet-scale resource-sharing frameworks such as volunteer computing assume established roles of resource providers and consumers rather than following a peer-to-peer model. This paper looked into how an adaptive overlay network can enable resource sharing across peers at the Internet scale by offering services while addressing the Internet’s challenges, such as computational resource scarcity, latency, and data loss. We designed Viseu, a blockchain framework to compose workflows from the service instances at the edge, sharing computational resources beyond organizational boundaries, with reward mechanisms and minimal overhead. As future work, we aim to deploy Viseu as an overlay framework for transfer learning workflows across multiple universities.

## Footnotes

1 Viseu is a city in Portugal’s Central Region with a rich history, often dubbed a cultural center. Viseu is surrounded by mountains and rustic towns.
